# Crosstalk Between the Oxidative Stress and Glia Cells After Stroke: From Mechanism to Therapies

**DOI:** 10.3389/fimmu.2022.852416

**Published:** 2022-02-25

**Authors:** Ganggui Zhu, Xiaoyu Wang, Luxi Chen, Cameron Lenahan, Zaixiang Fu, Yuanjian Fang, Wenhua Yu

**Affiliations:** ^1^ Department of Neurosurgery, Hangzhou First People’s Hospital, School of Medicine, Zhejiang University, Hangzhou, China; ^2^ Department of Neurosurgery, Second Affiliated Hospital, School of Medicine, Zhejiang University, Hangzhou, China; ^3^ Department of Medical Genetics, Second Affiliated Hospital, Zhejiang University School of Medicine, Hangzhou, China; ^4^ Center for Neuroscience Research, Loma Linda University School of Medicine, Loma Linda, CA, United States; ^5^ Department of Biomedical Science, Burrell College of Osteopathic Medicine, Las Cruces, NM, United States

**Keywords:** stroke, oxidative stress, astrocyte, microglia, therapies

## Abstract

Stroke is the second leading cause of global death and is characterized by high rates of mortality and disability. Oxidative stress is accompanied by other pathological processes that together lead to secondary brain damage in stroke. As the major component of the brain, glial cells play an important role in normal brain development and pathological injury processes. Multiple connections exist in the pathophysiological changes of reactive oxygen species (ROS) metabolism and glia cell activation. Astrocytes and microglia are rapidly activated after stroke, generating large amounts of ROS *via* mitochondrial and NADPH oxidase pathways, causing oxidative damage to the glial cells themselves and neurons. Meanwhile, ROS cause alterations in glial cell morphology and function, and mediate their role in pathological processes, such as neuroinflammation, excitotoxicity, and blood-brain barrier damage. In contrast, glial cells protect the Central Nervous System (CNS) from oxidative damage by synthesizing antioxidants and regulating the Nuclear factor E2-related factor 2 (Nrf2) pathway, among others. Although numerous previous studies have focused on the immune function of glial cells, little attention has been paid to the role of glial cells in oxidative stress. In this paper, we discuss the adverse consequences of ROS production and oxidative-antioxidant imbalance after stroke. In addition, we further describe the biological role of glial cells in oxidative stress after stroke, and we describe potential therapeutic tools based on glia cells.

## Introduction

Accounting for 9% of all deaths worldwide, stroke is the second leading cause of death following ischemic heart diseases (1). Hemorrhagic strokes caused by rupture of blood vessels in the cerebrum and ischemic strokes caused by occlusion of cerebral arteries constitute the two subtypes of stroke; the latter accounted for approximately 87% of all strokes (2). The high disability rate and poor prognosis that accompany stroke have greatly increased individual and societal burden. Therefore, stroke has become a major global public health and economic concern (3).

Ischemia-reperfusion and the release of damage associated molecular patterns (DAMPs) after stroke induce a series of pathological changes, including oxidative and nitrative stress, neuroinflammation, excitotoxicity, and apoptosis (4). Oxidative stress describes the state of imbalance between the systemic performance of reactive oxygen species and the ability of biological systems to detoxify active media or repair damage (5). Excess ROS interact with several of the pathological mechanisms described above and collectively mediates secondary brain injury after stroke (6–8).

Glial cells serve as the main supporting cells of the CNS. Recent studies have found that they are involved in the regulation of oxidative stress in the CNS. On one hand, Astrocytes and microglia are responsible for the imbalance of redox status after stroke (9). Glial cells have the potential to release large amounts of free radicals through various pathways (10, 11). The generated ROS promote the activation of inflammatory pathways in astrocytes (12), interfere with their transport of glutamate (13), and affect homeostasis of the intra- and extracellular microenvironment. Microglia are involved in the disruption of the blood-brain barrier after stroke and affect the post-stroke neural repair process (14). Conversely, astrocytes maintain high intracellular antioxidant concentrations to protect themselves from oxidative damage after stroke (15). Glutathione is the main antioxidant synthesized by glial cells and plays an important role in warding off oxidative damage (16). The intracellular Nrf2-ARE pathway is the main antioxidant pathway that synergistically regulates the redox status of glial cells and neurons (17).

Previous studies have mostly focused on the effects of oxidative damage on neurons within the brain, but few studies have explored the regulation of glial cells in oxidative stress after stroke. From the perspective of glial cells, this article aims to explain its influence on the occurrence and development of oxidative stress after stroke and to explore its interaction with other pathological mechanisms to provide new insight for future stroke treatments.

## The Role of ROS Under Physiological Conditions

As oxidants, physiological doses of ROS play an active role in various aspects of the body’s functional activities. From one hand, ROS contribute to the support of immune functions, such as enhancing macrophage phagocytosis and activating T cells (18). For another, ROS are engaged in the regulation of vascular cell proliferation and apoptosis. NO can maintain blood flow homeostasis in blood vessels (19). ROS promote the release of various germinal factors during wound repair and facilitate the repair process (20). During neurodevelopment, ROS is essential for synaptic plasticity and memory formation (21). In addition, researchers have found that ROS are also essential in muscle cell development and muscle remodeling (22). In conclusion, ROS can act as signaling molecules and are widely involved in maintaining homeostasis, metabolism, growth and differentiation *in vivo*.

## Oxidative Stress Generated by Glial Cells

### Mitochondria-Derived Oxidative Stress in Glial Cells

**Figure 1:**
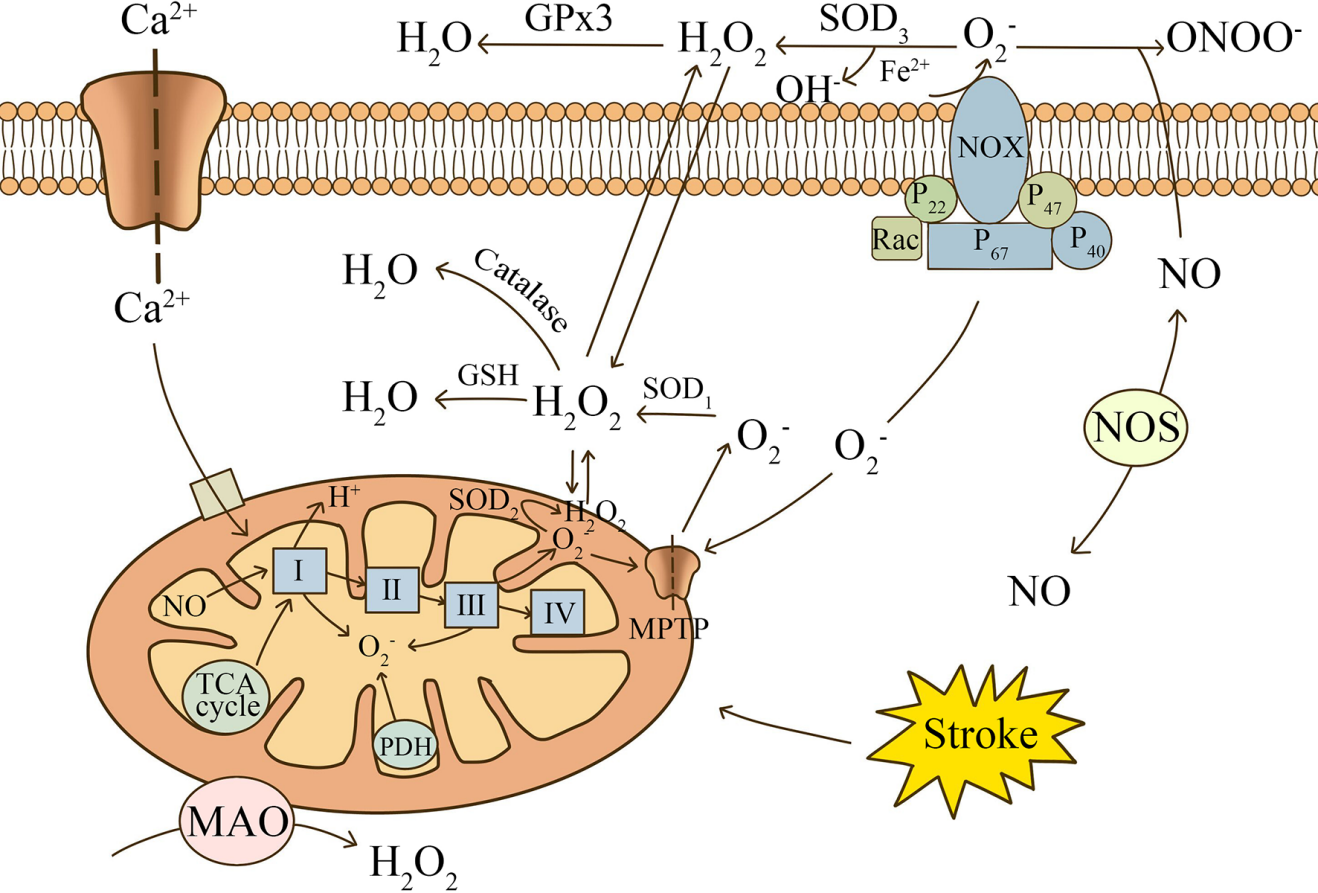
The main mechanisms of ROS production after stroke. Electrons released from the electron transport chain (ETC) react with O_2_ to produce superoxide. Mitochondrial complexes, oxidases in the matrix, and NADPH oxidase are involved in the release of ROS. Ca^2+^, NO, and succinate produced by the TCA cycle are important contributing factors. $O_{2}^{-}$ is catalyzed by SOD to produce H_2_O_2_, which can be decomposed into H_2_O and O_2_. NO can combine with $O_{2}^{-}$ to form ONOO^-^, causing nitrosative stress.

Mitochondrial metabolism in glial cells plays an important role in the regulation of redox status and biometabolism (see **Figure 1**) (10). Under physiological conditions, electrons released from the electron transport chain (ETC) react with O2 to produce superoxide $\left(O_{2}^{-}\right)$ .Free complex I in astrocytes is the major site of mitochondrial ROS (mROS) production (23). The metabolic state of the mitochondria determines the rate of $O_{2}^{-}$ production. The inhibition of respiration and the reduction in mitochondrial membrane potential following ischemia or hypoxia each induce electron leakage, which increases the output rate of reactive oxygen species significantly (24). Edward et al. (25) found that in a rat model of cerebral ischemia, succinate drove the rapid release of superoxide from complex I during reperfusion. Furthermore,higher ROS concentrations may result in prolonged activation of the mitochondrial permeability transition pore (MPTP) and inner membrane anion channel (IMAC). The opening of the above channels alters the changes in intra- and inter- mitochondrial redox homeostasis, leading to a surge of ROS. This ROS-induced ROS release (RIRR) mechanism highlights the mitochondria as the main source of ROS production (26). Superoxide dismutase(SOD) is present in large quantities in the mitochondria of glial cells, and it breaks down superoxide into oxygen and hydrogen peroxide (27). Catalase (CAT) and glutathione peroxidase (GPx) are also involved in the degradation of free radicals (28).

### NADPH-Derived Oxidative Stress in Glial Cells

The NOX family, especially NOX2 and NOX4, is highly expressed in glial cells (29–31). NADPH oxidase generates considerably more ROS than mitochondria and can release ROS out of the cell (32). Therefore, it is considered to be a key factor in the abnormal elevation of ROS after stroke. Translocation of the functional subunits, p47phox and p67phox, and the small GTPase, Rac1, is required for the activation of NOX2 (33). Toll-like receptors (TLRs) are important receptors on the glial cell surface that promote NOX activation. TLRs mediate phosphorylation of NOX functional subunits and oxidase activation mainly through MyD88-dependent and IL-1 receptor-associated kinase 4 (IRAK4)-dependent pathways (34, 35). The remaining surface receptors, including Complement receptor 3, Ionotropic P2X and metabotropic P2Y purinergic receptors, activate NOX mainly through the p38MAPK and ERK pathways (36, 37). Upon activation, NOX2 carries cytoplasmic electrons across the cell membrane and reduces extracellular molecular oxygen to $O_{2}^{-}$ . The released $O_{2}^{-}$ rapidly produces H_2_O_2_ with the participation of SOD_3_. H_2_O_2_ is highly lipid soluble and produces OH- through the Fenton reaction in the presence of iron, resulting in oxidative damage to most biological components (38). It is worth mentioning that mitochondria cross over with the NADPH oxidase pathway, increasing the likelihood of mitochondrial uncoupling, thus initiating a secondary surge of mitochondrial ROS (39).

### RNS Produced in Glial Cells

In addition to the oxygen radicals and derivatives that can be created after stroke as portrayed above, reactive nitrogen species (RNS) is also introduced in biological oxidation reactions. In the early stages of ischemia, NO released from endothelial NOS (eNOS) is usually considered to have a neuroprotective effect. Researchers have found that low doses of NO can cause vasodilation and improve blood supply to the penumbra region (40). In addition, Irina et al. found that eNOS-deficient mice had a higher likelihood of microcirculatory failure and death occurring after subarachnoid hemorrhage (41). However, studies have shown that glial cells release large amounts of NO under pathological conditions mainly through inducible NOS (iNOS) (42, 43). Lipopolysaccharide (LPS) and iron ions were found to induce iNOS expression in astrocytes and promote NO production (44, 45). Excess NO reacts with $O_{2}^{-}$ to form a strongly oxidizing peroxynitrite (ONOO^-^), leading to nitrosative stress (46). Mice experiencing transient or permanent cerebral ischemia have reduced infarct size and more preserved neurological function by knocking down iNOS gene expression or using NOS inhibitors (47, 48).Therefore, RNS may be a therapeutic target for the treatment of post-stroke ischemic injury.

## Oxidative Stress-Associated Pathological Processes After Stroke

### Oxidative Stress and Neuroinflammation

After cerebral ischemia, inflammatory reactions are initiated within the occluded blood vessels. Numerous cells, including astrocytes, are involved in the inflammatory process. ROS trigger nuclear transcription factors, such as nuclear factor kappa B (NF-κB), interferon regulatory factor 1, hypoxia-inducible factor 1, STAT3, activator protein 1 (AP-1), and p53, triggering the release of pro-inflammatory mediators (49). Interleukin-1β (IL-1β), tumor necrosis factor-α (TNF-α) and IL-6, are the main inflammatory factors that trigger and exacerbate the inflammatory response after stroke (50). Cell injury or necrosis release DAMPs, including high-mobility group box 1 (HMGB1), heat shock proteins (Hsps), and peroxiredoxin. DAMPs support excessive ROS production by activating NOX2 and NOX4 in inflammatory cells through pattern recognition receptors, CR_3_ and TLR_4_ (31). It is worth mentioning that microglia, driven by inflammatory factors, exhibit greater ROS production than neutrophils after stroke (51). Nuclear translocation of NF-κB plays a critical role in inflammatory cell-mediated responses to neuroinflammation and other injuries (52). Junghyung et al. (53) found that mROS regulates LPS-induced pro-inflammatory responses in microglia through the pathways of NF-κB and MAPK. In summary, oxidative stress and neuroinflammation are closely related, and together they contribute to the development of post-ischemic cerebral histopathological damage.

### Oxidative Stress and Excitotoxicity

Glutamate, the main neurotransmitter of the CNS, plays an essential role in message transmission, axon guidance, synaptic plasticity, and neuronal growth and development (54). After ischemia, free radicals are involved in the extracellular release of glutamate from nerve cells (55). Accumulation of glutamate in the extracellular space causes excitotoxicity (56). Glutamate can alter the osmotic gradient of the cell membrane, leading to excessive influx of water and Cl^-^ into the cell, causing neuronal swelling. In addition, glutamate activates receptors such as NMDA and AMPA, and the impaired Na^+^/K^+^ATPase function promotes the inward flow of calcium ions (57). Excess calcium ions accumulate in the mitochondrial matrix, impairing the mitochondrial electron transport chain and increasing ROS production (58). Furthermore, high extracellular glutamate concentrations inhibit cysteine transport into the C6 glia cells (59). As a raw material for glutathione (GSH) synthesis, a decrease in cystine implies a reduced antioxidant capacity, making neurons inevitably vulnerable to oxidative damage. Overall, the accumulation of glutamate, with abnormal changes in Ca^2+^ concentration, is very closely related to the increase in ROS after stroke.

### Oxidative Stress and Apoptosis

Programmed cell death, or apoptosis for short, is a strictly controlled process used to remove harmful cells in order to maintain cellular homeostasis (60). Excess free radicals can induce apoptosis by causing oxidative damage from lipid exposure, abnormal protein folding, and DNA breakage from multiple angles. Most of the peroxide-mediated apoptosis involves the activation of cysteinases. Additionally, H_2_O_2_ can induce increased mitochondrial membrane permeability, chromatin aggregation, and ultimately apoptosis by binding to death receptors and stimulating endoplasmic reticulum stress (60). When exposed to higher doses of H_2_O_2_, the tumor suppressor protein, p53, activates the transcription of pro-apoptotic genes (Bcl-2, Noxa) intracellularly and stimulates pro-apoptotic factors (Fas, DR-x) extracellularly to regulate apoptosis (61). Oxidative stress also upregulates the expression of MAPK and NF-KB pathways (62), and downregulates PI3K/Akt expression to trigger apoptosis (63). Additionally, the ROS-induced target protein, JNK, activates the expression of members of the Bcl family of pro-apoptotic proteins (64). The internal and external pathways of apoptosis are coordinated, as they both ultimately activate caspase proteins, which together play a role in causing programmed cell death (65).

## Astrocyte in Stroke

### Reactive Astrogliosis and Glial Scar in Oxidative Stress

**Figure 2:**
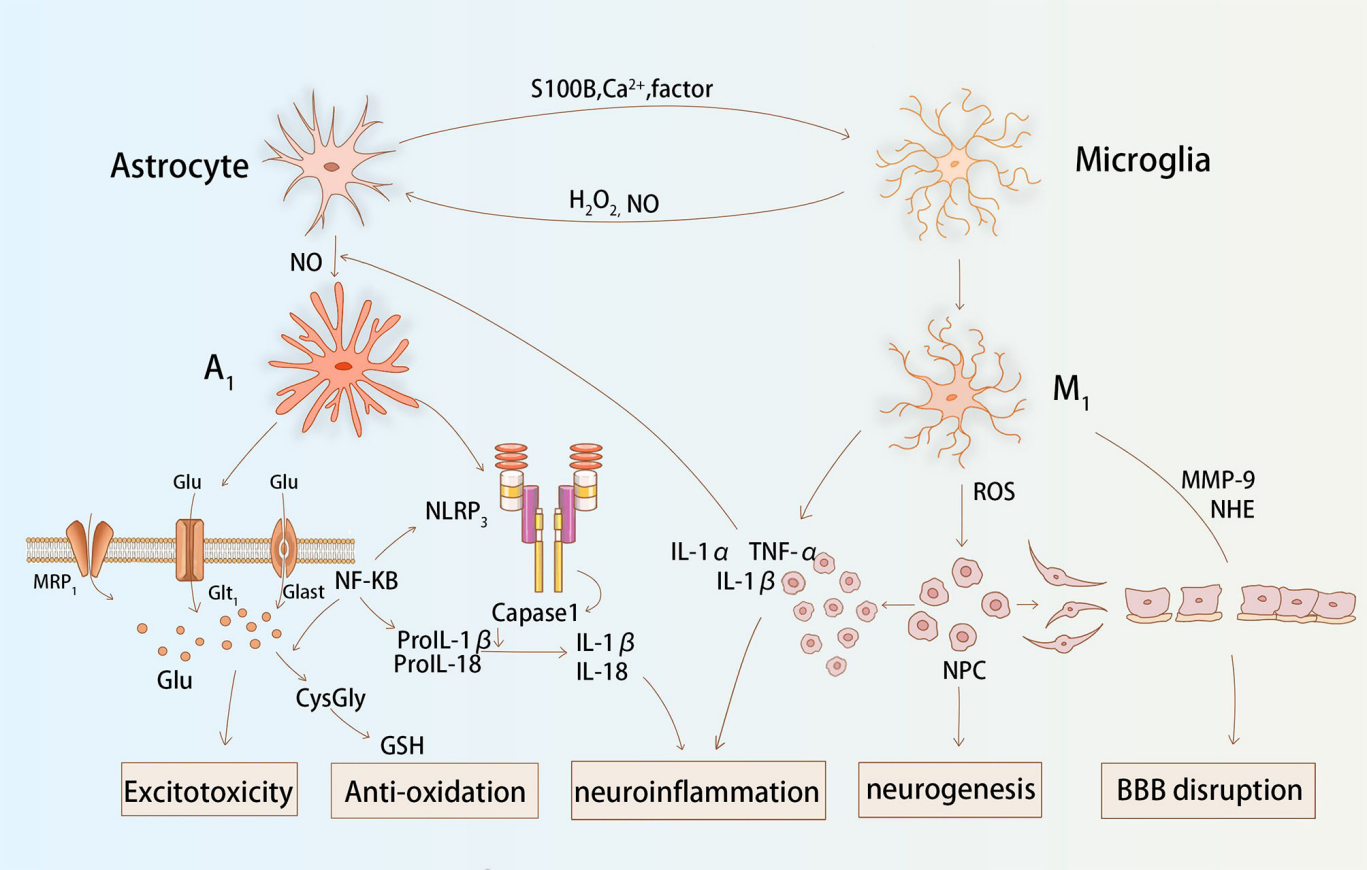
Astrocytes and microglia interact and function together during stroke. Astrocytes release Ca^2+^, S100B, and cytokines that affect the redox state of microglia. The production of H_2_O_2_ and NO by microglia facilitates the activation process of astroglial. Glial cells affect glutamate transport, inflammatory factor secretion, matrix protease activation, and antioxidant production *via* ROS, which together cause post-stroke excitotoxicity, neuroinflammation, BBB destruction, and neurogenesis.

The normal functions of astrocytes in the maintenance of the CNS have been well documented (9, 66). In the absence of cerebral blood flow, astrocytes swell, proliferate rapidly, and migrate, and is coupled with increased cell retention of glial fibrillary acidic protein (GFAP) and vimentin (Vim), which signals the onset of reactive astrogliosis (67). A growing number of studies report that certain oxidative stress molecules, such as NO, H_2_O_2_, are important in the induction of reactive astrogliosis and glial scar formation (see **Figure 2**) (68). Amita et al. (69) observed that GFAP and VIM expression was upregulated in astrocytes after exposure to H_2_O_2_, and that the intervention of curcumin served to mitigate this tendency. Swarnkaret et al. (70) found that increased ROS upregulated GFAP expression in C6 astrocytes after mitochondrial inhibition with rotenone. Several researchers have shown increased expression and secretion of chondroitin sulfate proteoglycan (CSPG) in an *in vitro* reactive astrocyte model they designed, which was shown to have an inhibitory effect on neurite growth in axon guidance site assays (71). Excessive astrocyte proliferation combined with pericytes is involved in the formation of glial scar, which is thought to act as a mechanical barrier to the recovery of neurological function (72).Nrf2 is a major antioxidant pathway in astrocytes, and researchers found that a significant reduction in the number of reactive astrocytes in the CA1 region of the hippocampus was related to activation of the Nrf2 pathway following ischemia and hypoxia (73). Liu et al. found that mice lacking Nrf2 had reactive glial proliferation after permanent distal middle cerebral artery occlusion and elevated expression of the water regulatory protein aquaporin 4 levels, and significantly larger infarct size compared to controls (74).Sustained attenuation of reactive glial proliferation in astrocytes and microglia is highly correlated with Nrf2, providing late neuroprotection to ischemic and hypoxic neurons (73). In summary, redox status influences the state of reactive astrocytes and glial scar formation, in which Nrf2 plays an important role.

### Astrocytic Oxidative Stress and Neuroinflammation in Stroke

It has been found that glial cells are frequently present in both oxidative stress and inflammatory processes in CNS. ROS activate reactive astrocytes and microglia, stimulate multiple inflammatory signaling pathways, and consistently release large amounts of inflammatory factors (75). NLRP3 inflammasome is the protein complex that has received the most attention in the NLRP subfamily. It has been noted that mROS is involved in ethanol-induced NLRP3 inflammasome activation in astrocytes and that it recruits procaspase 1 (76). It is hydrolyzed to caspase 1 to promote the release of IL-1β and IL-18, which ultimately induces inflammation. Not coincidentally, NADPH oxidase-derived H_2_O_2_ is involved in the induction of the inflammatory cascade reaction that occurs in astrocytes with LPS/IFNγ, causing glial cells to express different cytokines, namely IL-1, IL-6, and TNF (77). In *in vitro* experiments, researchers pre-treated cultured primary astrocytes with oxygen and glucose deprivation. They found that astrocytes secreted significantly higher levels of IL-1β and TNF-α when re-exposed to temporal oxidative stress (78). Moreover, glial cells exposed to inflammation also release ROS to increase the level of oxidative stress. Studies have shown that stimulation of astrocytes with TNF-α and IFN-γ leads to mild upregulation of IL-6 and iNOS and the production of harmful free radicals (79). IL-1 is also involved in the activation of iNOS and stimulates the release of high levels of NO. Nrf2 is a major antioxidant gene in astrocytes. Researchers found that knockout Nrf2 mice secreted more TNF-α, IL-1β, IL-6, and MMP9 *via* the NF-κB pathway in astrocytes than in controls (80).During the later stages of stroke, astrocytes also secrete factors, such as transforming growth factor-beta (TGF-β), that exert an active anti-inflammatory effect (81).

Overall, ROS can influence the inflammatory response of glial cells. Similarly, inflammatory factors can regulate the redox status of glial cells. The interaction between glial cell ROS and neuroinflammation is a common pathological basis for several CNS diseases.

### Astrocytic Oxidative Stress and Excitotoxicity in Stroke

Astrocytes indirectly influence redox levels by regulating extracellular glutamate concentrations mainly through two specific transporter proteins (GLAST: glutamate/aspartate transporter protein and GLT-1: glutamate transporter protein 1) (82). In experiments using GLAST/GLT-1 knockout animals, slower glutamate clearance rates were found in the knockout animals compared to controls, and neuronal deformation was also observed in the hippocampal CA1 region (83). After conducting *in vitro* experiments, researchers found that GLAST/GLT-1 redistribution occurred in astrocytes after oxygen and glucose deprivation, causing a consequent impairment of glutamate transport (84). The accumulation of extracellular glutamate stimulates Ca^2+^ channels to stay open for an extended duration, resulting in a large influx of Ca^2+^. NMDA receptors or AMPA receptors are activated by Ca^2+^, and generate large amounts of ROS (57).

Relatively, the transport of glutamate by astrocytes is also conditioned by the redox state of the CNS (84, 85). In a rat model of cerebral hemorrhage, researchers observed a decrease in GLAST and GLT-1 expression at 6 hours and 72 hours, respectively, which was accompanied by a massive amount of ROS production (86). Antioxidants such as hydrogen sulphide (H_2_S) maintain normal glutamate metabolism by enhancing glutamate uptake by astrocytes pre-treated with H_2_O_2_ (87). In the study by Matos et al. (88), Aβ inhibited GLAST and GLT-1 in a non-competitive manner to reduce glutamate intake, with a cascade of oxidative stress and MAPK signaling pathways involved. In astrocytes, ROS is also involved in the secretion of glutamate. A study found that ROS production in rat hippocampal astrocytes cultured with ethanol stimulated glial cell cytostasis and increased glutamate secretion, leading to hippocampal neurotoxicity (89). Paralleled with ROS, the RNS plays a similar role. The project by Tomoaki et al. confirms that there is a prominent relationship between NO production and enhanced cytokine-induced calcium-dependent glutamate release in astrocytes cultured *in vitro* (90). Conversely, the effects of the remaining free radical components on glutamate transport and metabolism in astrocytes have seldomly been reported and still warrants exploration.

### Astrocytic Oxidative Stress and Synthetic GSH in Stroke

Glutathione, as an important oxidative detoxifier in the body, plays an important role in protecting cells from ROS. GSH exerts its antioxidant effect when oxidized by hydrogen peroxide and lipid peroxides to Glutathione disulfide (GSSG) (91). Astrocytes appear to be important in GSH metabolism as they can deliver more GSH compared to microglia, oligodendrocytes, and neurons (92). Researchers found that in mice with brain hemorrhage, heme induced peroxynitrite production, causing oxidative damage. This damaging effect was shown to be associated with GSH depletion in astrocytes, and the toxic response was attenuated by exogenous GSH supplementation (93). Guo et al. (94) showed that the AMPK-PGC-1α signaling axis positively stimulates γ-glutamylcysteine synthetase expression in astrocytes, and it increases the rate of GSH synthesis. The antioxidant effects of dehydroascorbic acid (DHA) are widely known, and studies have found that DHA increases the rate of glutathione production in primary astrocytes, thereby protecting astrocytes from H_2_O_2_-induced cytotoxicity (95). GSH synthesis is also regulated by the inflammatory response, and *In vitro* studies have demonstrated that IL-1β stimulates astrocyte upregulation of GSH synthesis *via* the NF-κB pathway (96). Besides, astrocytes also release GSH precursors (CysGly) that can be used for *de novo* synthesis of GSH in neurons, indirectly increasing oxidative defense capacity (97). Members of the multidrug resistance protein (MRP) family, mainly MRP1, reportedly mediate the extracellular release of astrocytic GSH and similarly transmit the efflux of the strongly oxidative GSSG (98). It has been demonstrated that the antioxidant, alpha-tocotrienol (TCT), may exert neuroprotective effects after stroke by inhibiting MRP1-mediated GSSG release (99). Johannes et al. (100) showed that high concentrations of MK571 (MRP1 inhibitor) could only achieve 63% inhibition of GSH release from astrocytes. This suggests that there are other protein channels involved in GSH transport and that other MRP family members are candidates (92).

### Astrocytic Oxidative Stress and Nrf2-ARE Pathway in Stroke

**Figure 3:**
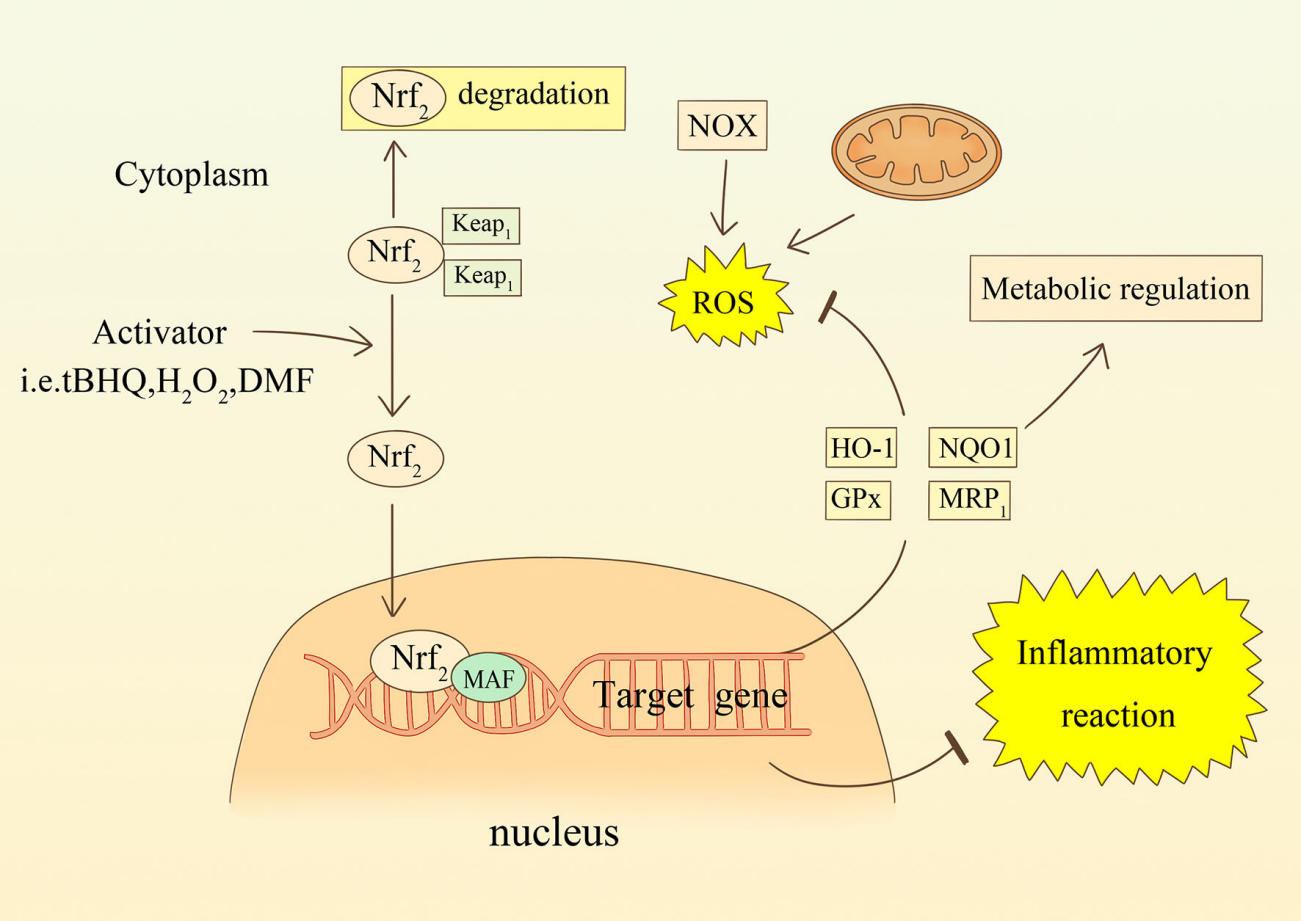
The relationship between oxidative stress and the Nrf2 signaling pathway in astrocytes. ROS or compounds stimulate Nrf2 transcription into the nucleus and participate in the transcription of target genes. increased synthesis of enzymes such as HO-1, NQO1, GPx, and MRP1 counteract oxidative stress and regulate metabolism. Nrf2 is also involved in regulating inflammatory responses.

The Nrf2-ARE signaling axis is an essential endogenous antioxidant pathway (101). Nrf2 is an inducible transcription factor that promotes the expression of cytoprotective proteins. ARE is an antioxidant response element present in a variety of functional enzymes, such as HO-1, NADPH-quinone oxidoreductase (NQO1), glutathione synthase and MRP1 (see **Figure 3**) (102). Astrocytes have a powerful capacity to cope with oxidative harm and are resistant to oxidative stress because the Nrf2-ARE pathway is extremely stable within them (103). A study using mixed primary cultures showed that tert-butylhydroquinone (tBHQ)-induced ARE activation effects were highly selective for astrocyte subpopulations, not neurons (104). Furthermore, in an *in vitro* model of cerebral ischemia, increased expression of Nrf2 by tBHQ prompted astrocytes to secrete the anti-inflammatory cytokine, IL-10 (78). Another experiment using sub-toxic doses of H_2_O_2_ to induce Nrf2 activation also demonstrated that astrocytes are critical loci (105). In some models of permanent cerebral ischemia(pMCAO), Nrf2 and protein expression levels of HO-1 and SOD were upregulated in the ischemic cortex within 24 hours of the intervention (106). Cytoprotective proteins downstream of Nrf2, such as HO-1 and NQO1, were significantly increased in peri-infarct tissue samples ranging from 1.8 to 3.6-fold shortly after ischemia, but not in Nrf2- mice (107). Xie et al. found that the use of Leonurine increased the expression level of Nrf-2 mRNA, and decreased the level of ROS in pMCAO of male rats (108). Dimethyl fumarate (DMF) specifically activates Nrf2 and exerts neuroprotective effects against ischemic brain injury *via* the Nrf2/HO-1 pathway. Researchers found that DMF significantly reduced the area of brain swelling, the incidence of neurological deficits, and the quantity of activated microglia and macrophages (109, 110). Moreover, Nrf2 is also involved in regulating the metabolism of hemoglobin and iron, and plays a role in avoiding neurological damage after intracerebral hemorrhage (111). In a Parkinson’s disease(PD) mouse model, Nrf2 knockout mice are more prone to PD-like damage, and this toxic response is mitigated by overexpression of Nrf2 in astrocytes (112). Using transgenic mice overexpressing Nrf2 crossed with amyotrophic lateral sclerosis mice, the researchers found that mice with the specific gene had a delayed onset of disease and longer duration of survival (113). The Nrf2 pathway is therefore an effective and promising therapeutic target in the regulation of oxidative and reductive homeostasis, whether in neurodegenerative disease or in stroke.

## Microglia in Stroke

### Microglia Activation After Stroke

Microglia are the resident immune cells of the CNS. They are rapidly activated and migrate to the lesion area in response to harmful stimuli, such as infection, stroke, trauma, tumors, etc. Activation of microglia is a key part of the cerebral defense system. Microglial activation can be detected in the ischemic core and in the peri-infarct area 24 hours after cerebral ischemia (114). To identify activated microglia more precisely, Iba1, IB4, F4/80, CD11b, and CD68 have been defined as surface markers of activated cells, and increased CD11b expression corresponds to the severity of microglial activation (115). The researchers induced high CD11b expression in mouse BV-2 microglia by stimulating them with bacterial LPS and other harmful molecules, such as IL-1β, IL-12, and double-stranded RNA (poly(IC)), but this effect was blocked by antioxidants, suggesting that ROS are involved in microglial activation (116). Another study also corroborated that inhibition of ROS significantly prevented cell proliferation and microglial activation (117). According to Palwinder’s description, microglial activation due to the pro-inflammatory factor, IL-1β, was inhibited in primary rat glial cultures when an NADPH oxidase inhibitor (apocynin) was introduced. When NADPH oxidase activator, such as arachidonic acid, was introduced into the system, the rate of microglia proliferation was accelerated (118). The above evidence suggests that ROS, mainly hydrogen peroxide from NADPH oxidase, may be a key player in the activation and proliferation of microglia after stroke.

### Oxidative Stress and Neuroinflammation in Microglia After Stroke

Different stimulating factors, periods of action, and environmental variation stimulate microglia to undergo polarization into different phenotypes: M1 (classical pro-inflammation) and M2 (alternative protection) (119, 120). INOS and arginase-1 are often considered accurate markers for the characterization of M1 and M2 microglia, respectively (121). Siddharama et al. (77) found that NADPH oxidase in rat microglia was rapidly activated by LPS and IFNγ and that it released large amounts of ROS, which induced iNOS and NO production. However, when they transfected microglia with a mutant form of the p47 phox subunit of NADPH oxidase, the expression of both ROS and iNOS was inhibited. This suggests that the pro-inflammatory response of microglia requires the involvement of functional NADPH oxidase and that ROS is an important mediator in the activation of MAPK and NF-κB. Furthermore, the researchers found that H_2_O_2_ was the ROS that promoted iNOS expression in LPS-pre-treated microglia, although this effect could not be triggered when H_2_O_2_ was applied alone (122). In *in vivo* and *in vitro* stroke models, it was found that NOX2-mediated oxidative stress in microglia induces the onset of inflammatory responses after ischemia-reperfusion. Inhibition of NADPH oxidase using apocynin resulted in significant inhibition of IL-6 release levels from microglia (123). IL-4 is commonly used to induce activation of M2 microglia and expression of arginase. Rotenone inhibits IL-4-induced activation of the M2 phenotype in microglia and expression of arginase, as described by Annette et al. (124). Furthermore, they found that IL-4 reduced LPS-stimulated IL-6 and TNF-α secretion, and that the addition of rotenone attenuated this effect. By specifically knocking out p47 phox subunit, the researchers discovered that microglia from p47 phox-/- mice overexpressed IL-4 and arginase 1 compared to wild-type mice, a phenomenon also verified in mice treated with NADPH oxidase inhibitors (125). Nrf2 plays a role in facilitating the transition of microglia to M2. The researchers found that in a model of Parkinson’s disease, Nrf2-deficient mice exhibited an increased M1 phenotype, as well as a decreased M2 phenotype (126). We can conclude that in the presence of ROS, microglia may tend to polarize toward M1 and reduce the activation of M2 to exert their vital role in inflammation.

Notably, in recent years, M2 microglia exhibit three subtypes, M2a, M2b, and M2c, depending on the period of stimulation (127).The role of ROS in regulating these three subtypes in ischemia should be further investigated.

### Oxidative Stress and Neurogenesis in Microglia After Stroke

Although stroke results in massive neuronal death, a mounting number of studies are finding compensatory neurogenesis as an important mechanism for neural repair and maintaining brain plasticity after injury (128, 129). The subgranular zone (SGZ) of the dentate gyrus and the subventricular zone (SVZ) of the lateral ventricles are commonly believed to be areas of neurogenesis (130). The aggregation of chemotactic microglia to neurogenic ecological sites is fundamental in exerting their function. Lelli et al. (33) found that NOX2 mediates these processes through colony-stimulating factor 1 receptor (CSF-1R) and vascular endothelial growth factor receptor 1 (VEGFR1). Estrada et al. (131) found that exogenous addition of H_2_O_2_ promoted neural progenitor cell (NPC) differentiation and increased the number of oligodendrocytes. Furthermore, microglia remove redundant neurons through phagocytosis. Wakselman et al. (132) demonstrated that CD11b integrin and DAP12 immunoreceptors regulate ROS production in microglia and mediate apoptosis of neurons in the developing hippocampal region. Another study demonstrated that microglia produce $O_{2}^{-}$ and subsequently, physiologically reject Purkinje fibers (133). However, the effect of microglia on neurogenesis is biphasic. After ischemia, Nrf2 levels were significantly reduced in pyridoxine-deficient mice, and neurotrophic factor (BDNF) showed the same trend. This suggests that oxidative stress reduces the regenerative capacity of neurons in the hippocampal region by reducing the secretion of trophic factors (134). In an experiment with co-culture of NPCs and microglia, researchers found that microglia-derived ROS inhibited NPC proliferation *via* glycogen synthase 3, thereby affecting neural recovery in mice with Parkinson’s disease (135). This was associated with inhibition of the wnt/β-catenin signaling pathway. The secretion of IL-1β by microglia in another experiment activated the p53 gene in NPC by promoting the production of ROS, which stalled the NPC cell cycle and hindered their differentiation process (136). Besides, NO released from microglia exhibits an antiproliferative effect on NPC in the SVZ region (137). Compared with the control group, the release of NO was significantly reduced and the proliferation of NPC was remarkably enhanced in the knockdown iNOS medium. Indeed, the specific relationship between microglia-derived ROS and neurogenesis remains unclear, which requires more research in the future.

### Oxidative Stress and Blood Brain Barrier in Microglia After Stroke

The blood-brain barrier (BBB) is a physical barrier formed by endothelial cells (EC) relying on tight junctions (TJ), and is essential for maintaining CNS homeostasis. After stroke, BBB disruption leads to inflammatory cell infiltration and release of toxic factors, increasing disability and mortality. There is evidence that ROS released from activated microglia inevitably damage the BBB (138). In the early stages, ROS induce EC death and drive BBB destruction. EC structures can be maintained *via* inhibition of ROS through apocynin (14). The researchers found that NOX4 was hyper-expressed in microglia in the ICH model, resulting in remarkable downregulation of multiple TJ protein, including ZO-1, occludin, and claudin-5 (139). After knockdown of NOX4, tight junction proteins were upregulated, brain edema was significantly improved, and BBB integrity was restored. At later stages of BBB disruption, ROS activated MMP-9, which degrades collagen and laminin in the basement membrane and disrupts basement membrane integrity. Usatyuk et al. (140) found that ROS cause lipid peroxidation, leading to an increase in 4-hydroxynonenal (NHE). NHE alters BBB permeability by modulating intercellular adhesion, but also diffuses to distant compartments to impair cellular function. Besides, after stroke, iNOS expression is increased in M1 microglia. Both NO and peroxynitrite can lead to BBB destruction (141). The use of NOS inhibitors prevents MMP activation and attenuates BBB damage. Not specifically, ROS can induce chemotaxis in leukocytes to migrate to CNS, and their released products further degrade the BBB (142). In conclusion, microglia-derived ROS have a very important role in regulating the integrity of the BBB, and will likely be a new direction for future treatment.

## Glial Cell-Related Neural Repair After Stroke

A growing number of studies have demonstrated that the adult CNS remains plastic after damage. Glial cells are important players in many processes of neural repair or regeneration. First, glial cells, especially M2 microglia secrete the anti-inflammatory factor IL-10, which counteracts the development of neuroinflammation after stroke and protects endothelial cells from oxidative stress injury (143). IL-4 downregulates the release of ROS or RNS, thereby limiting BBB infiltration (144). Secondly, glial cells can promote stroke repair by promoting angiogenesis and BBB repair. ROS promotes the release of vascular growth factor (VEGF) (145). VEGF, administered late after stroke, promotes angiogenesis in ischemic strokes (146). Although MMP-9, which has a destructive effect in the early phase, is upregulated in the peri-infarct cortex 7-14 days after stroke and shows a angiogenesis-promoting function (147). Moreover, sonic hedgehog released from astrocytes promotes the upregulation of tight junction proteins between capillary endothelial cells to promote the restoration of BBB integrity (148). Thirdly, glial cells play an important role in promoting synaptogenesis and plasticity. Glial cell-derived cholesterol serves as a raw material for synaptogenesis, promotes synaptogenesis and regulates myelin formation after stroke (149). Studies have reported that various molecules secreted by glial cells, such as TNF-α, thrombospondin, hevin have the ability to repair synaptic dysfunction after stroke (150). Beyond that, microglia and astrocytes secrete various nutrients and growth factors such as BDNF, nerve growth factor (NGF), and basic fibroblast growth factor (bFGF), which are useful for neural repair and plasticity (151, 152). In fact, the role of ROS and oxidative stress on brain tissue repair after stroke is still poorly understood, and most of the current studies focus on the aspects that promote brain injury; therefore, further studies on the specific mechanisms of ROS and oxidative stress and cell repair after stroke are needed in the future.

## Crosstalk Between Astrocytes and Microglia in Oxidative Stress After Stroke

Both microglia and astrocytes are active participants in the post-stroke pathological process. On one hand, microglia respond to injury earlier than astrocytes and promote astrocyte activation. The surge of ROS after stroke promotes the release of multiple inflammatory mediators from microglia (53). IL-1α and TNF-α induce astrocyte activation and promote their proliferation (153). IL-1β mediates the release of NO from astrocytes, which induces neurotoxicity (154). IL-1β also inhibits the ability of astrocytes to transport glutamate, inducing excitotoxicity in neurons (155). In addition, in Parkinson’s disease, H_2_O_2_ produced by microglia can act as a direct signal to regulate astrocyte proliferation, which is associated with STAT1/3 phosphorylation (156). In a model of chronic neuroinflammation, the P2Y6 receptor is involved in the release of NO from microglia, and it promotes astrocytic apoptosis to prevent excessive proliferation (157). Conversely, astrocytes are also involved in regulating the redox status of microglia. The Ca^2+^ modulated protein, S100B, is released by astrocytes. At physiological concentrations, S100B acts as a trophic factor and exerts neuroprotective effects (158). At higher concentrations, researchers found that S100B stimulates ROS release from microglia and activates p38 and JNK kinases, which mediate NO production (159). Furthermore, Min et al. (160) found that astrocytes produce a soluble heat-intolerant factor that regulates the redox state of microglia. They found that the level of ROS was significantly reduced after treating microglia with astrocyte culture conditioned medium for 6 and 12 hours. This inhibitory effect was associated with nuclear translocation of Nrf2 and promoted the expression of the antioxidant enzyme, HO-1, in an ARE-dependent manner. Moreover, astrocytes contribute to the regulation of microglial cell function. H_2_O_2_-exposed astrocytes activate the STAT6-COX2 pathway, which regulates the release of TNF-α from microglia through the release of PGE2 and PGI2 (161). Most of the current research exploring crosstalk between microglia and astrocytes has focused on the interaction of immune function, but further studies on the role of oxidative stress in each are necessary.

## Potential Therapeutic Targets

It is widely recognized that oxidative stress mechanisms following stroke is an important cause of secondary brain damage. Therefore, inhibition of ROS/RNS production and enhancement of its scavenging efficiency are currently considered viable antioxidant strategies. **Table 1** summarizes the relevant drugs and their main mechanisms of action.

**Table 1:** Approaches to post-stroke oxidative stress.

Categories	Drug	Mechanism	Reference
**Inhibit ROS generation**	Apocynin	inhibits the migration of the p47 (phox) subunit, inhibit inflammatory, decrease apoptosis	(162–167)
**Scavenge ROS**	Edaravone	reduces H_2_O_2,_ and lowers overall oxidative stress levels	(168–171)
	Melatonin	scavenges free radicals, enhances antioxidants capacity, inhibits inflammation and NADPH oxidase	(172–174)
	Sirtuin 1	upregulates SOD2 in astrocytes	(175)
	Ebselen	attenuates oxidative damage	(176)
	Resveratrol	increases HO-1 expression and extracellular GSH content	(177)
	Sulforaphane	activates the Nrf2 pathway and downstream proteins	(178)
**Endogenous antioxidants**	Vitamins	scavenges O_2-_,peroxyl radicals,down-regulates the activity of NADPH oxidase	(179)
	Desferrioxamine	Inhibits the Fenton reaction by chelating Fe^2+^	(180, 181)
	Ngb1	acts as a reductase, protects mitochondria, regulate calcium levels	(182, 183)
**Mitochondria-targeted protective agents**	Mitoquinone	reduces mROS generation, inhibits M1 microglia activation	(174, 184–188)
**Epigenetic factors (microRNA)**	miR-424	increases levels of SOD, MnSOD, and Nrf_2_ expression, inhibits microglia activation and inflammatory factor production	(189)
	miR-152-3p	reduces production of reactive oxygen species and apoptosis	(190)
	miR-652	inhibits of NOX_2_ expression and ROS production	(191)
	miR-124	affects the activation of the M2 phenotype	(192, 193)
	miR-421-3p	Inhibits expression of pro-inflammatory factors and NF-κB activation in microglia	(194)
	microRNA-181a (antagomir)	Suppresses microglia activation, leukocyte infiltration, and endoplasmic reticulum stress	(195)

### Inhibition of ROS-Producing Enzymes

The role of NADPH oxidase in mediating oxidative stress after stroke is well established. Apocynin inhibits NADPH oxidase by inhibiting the migration of the p47 phox subunit to the membrane, thus exerting its antioxidant capacity (162). Apocynin-treated microglia exhibit reduced NADPH oxidase activity, decreased ROS production, and reduced necrosis of SH-SY5Y neurons (163). The study demonstrated that after 24 hours of ischemia-reperfusion, mice in the Apocynin-treated group had approximately 50% less infarct area than the wild group (164). Besides, Apocynin significantly inhibits the development of the inflammatory cascade response and decreases the level of apoptosis (165). It is worth mentioning that Apocynin can also act as a non-specific free radical scavenger to reduce oxidative stress (166). Meanwhile, melatonin, an endogenous antioxidant, prevents the complete assembly of NADPH oxidase in microglia. It inhibits the production of superoxide and its derivatives, which may be related to the PI3K/Akt signalling pathway (167).

### Scavenge ROS

Edaravone is a synthetic free radical scavenger whose conferring neuroprotective effects have been demonstrated in animal studies (196). *In vitro*, researchers have demonstrated that edaravone protects neurovascular unit components, including neurons, glial cells, and endothelial cells, from oxidative cell death (168–170). In a randomized clinical trial for acute ischemic stroke, the edaravone treatment group showed higher neurological outcomes at day 90 post-onset (171). However, whether edaravone can modulate inflammation is controversial, and this warrants further exploration in the future.

Melatonin is a neuroendocrine hormone that protects normal cell function by scavenging free radicals, enhancing antioxidants, and inhibiting inflammation (172). We found that ROS and superoxide production were reduced and DNA damage was significantly alleviated in C6 astrocytes treated with melatonin (172, 173). Additionally, melatonin has been demonstrated as a targeted mitochondrial protector, reducing mROS formation (174).

Furthermore, a multitude of compounds exist that exert antioxidant effects and protect the nervous system, such as Sirtuin 1 (175), Ebselen (176), Resveratrol (177), and Sulforaphane (178).

### Endogenous Antioxidants

Vitamin C (ascorbic acid) scavenges free radicals produced by normal cellular metabolic processes and also down-regulates the activity of NADPH oxidase, reducing the generation of ROS (179). Nevertheless, fewer studies have been conducted to clarify whether the antioxidant properties of the vitamin are related to glial cells. Hypoxia-inducible factor 1 (HIF-1) plays an integral role in inducing cells to build tolerance to hypoxia (197). Following ischemia, HIF-1 and many of its target genes, such as erythropoietin (EPO) and VEGF, are increased and exert neuroprotective effects (198). Deferoxamine is an inducer of HIF-1 and its mediated antioxidant effect may be related to the inhibition of the Fenton reaction by chelating Fe^2+^ (180). Clinical trials conducted by Mònica et al. confirmed the effectiveness and safety of desferrioxamine in the treatment of ischemic stroke (181).Neuroglobin protein (ngb1) is a member of the bead protein family and its initiation process is regulated by transcription factors, such as HIF-1 and VEGF (182). Animal experiments have demonstrated that sub-nanomolar concentrations of Ngb1 prevent ROS accumulation, reduced antioxidant enzymes, and reduced expression of apoptotic proteins in astrocytes after exposure to H_2_O_2_ (183).

### Mitochondria-Targeted Protective Agents

Mitoquinone (MitoQ), a mitochondria-specific antioxidant, has the advantage of being able to penetrate the mitochondrial phospholipid layer and accumulate rapidly, thereby eliminating ROS at the source (184). The researchers observed that carbon nanoparticles C3, through photosensitizing effects and depolarization of mitochondrial membrane potential, caused astrocytes to produce large amounts of ROS (174). This effect was significantly inhibited when glial cells were protected by MitoQ. Furthermore, it has been observed in ICH models that MitoQ inhibits M1 microglia activation and promotes the M2 phenotype shift, which also contributes to the reduction of brain edema and BBB destruction (185). Mice treated with 4 mg/kg MitoQ after TBI exhibited increased activity of multiple antioxidant enzymes (including SOD and GPx) and attenuation of neurological deficits, an effect likely mediated through activation of the Nrf2/ARE pathway (186). A recent clinical study showed that high oral ingestion of Mitoquinone improved endothelial function, antioxidant enzyme activity, and exercise tolerance in patients with peripheral arterial disease (187). Despite its powerful and rapid antioxidant capacity, the safe dose range of MitoQ has not been defined (188).

### Epigenetic Factors (microRNA)

The intron DNA encodes the production of MicroRNA, which binds to mRNA, and either accelerates, slows, or blocks the translation process of mRNA, thereby regulating different aspects of the pathophysiological process of stroke (199). Studies have shown that treatment with miR-424 mice after 24 hours of acute cerebral ischemia exhibited increased levels of SOD, MnSOD, and Nrf2 expression (189). MiR-424 also limits the development of cerebral infarct volume by inhibiting microglial activation and inflammatory factor production. Besides, miR-152-3p agomirs (190) and miR-652 (191) also showed different levels of antioxidant capacity. A certain miRNA expressed only in the CNS, such as miR-124, can reduce neuronal death after MCAO by affecting the activation of the M2 phenotype in microglia (192, 193). Finally, miR-421-3p (194) and miR-181a (195) could also protect neurological function by modulating the post-stroke inflammatory response. These studies suggest that the use of miRNAs offers new insights into mitigating neurological impairment and promoting brain recovery after stroke.

## Conclusion and Perspectives

In summary, the redox status of the central nervous system influences the secondary damage and repair of brain tissue after stroke. Glial cells play a key role in maintaining the balance of oxidation and antioxidation in the CNS under physiological or pathological conditions. On one hand, neuroglia can act as a major source of ROS, causing oxidative damage and mediating secondary damage, such as neuroinflammation, excitotoxicity, and blood-brain barrier disruption. On the other hand, ROS affect the phenotype of glial cells, such as activating astrocytes and promoting polarization of microglia. Thus, ROS is an important mediator for glial cells to perform their functions. Glial cells are involved in the neural repair process after stroke, including limiting the spread of diseased tissue, enhancing antioxidant capacity, and participating in neurogenesis. In addition, there are associations between various glial cells that jointly mediate the pathophysiological processes after stroke. Recently, studies on antioxidant therapy have started to focus on the role of glial cells. These studies have been conducted from the perspective of inhibiting ROS production or accelerating ROS clearance, and some progress has been made. However, considering the different roles played by glial cells at various times, studies should be individually designed. Future studies can further explore the specific mechanisms of glial cell-mediated oxidative stress and the functional differences between different glial cell types, which would be very promising targets in the treatment of CNS diseases.

## Author Contributions

GZ and XW were responsible for the study concept, design, and writing of the first draft. LC, CL, ZF, YF, and WY participated in the literature collection. All authors agreed to approve the final manuscript.

## Funding

This study was supported by financial assistance from the Department of Neurosurgery, Hangzhou First People’s Hospital, Zhejiang University School of Medicine.

## Conflict of Interest

The authors declare that the research was conducted in the absence of any commercial or financial relationships that could be construed as a potential conflict of interest.

## Publisher’s Note

All claims expressed in this article are solely those of the authors and do not necessarily represent those of their affiliated organizations, or those of the publisher, the editors and the reviewers. Any product that may be evaluated in this article, or claim that may be made by its manufacturer, is not guaranteed or endorsed by the publisher.
